# A Frequency-Domain Adaptive Matched Filter for Active Sonar Detection

**DOI:** 10.3390/s17071565

**Published:** 2017-07-04

**Authors:** Zhishan Zhao, Anbang Zhao, Juan Hui, Baochun Hou, Reza Sotudeh, Fang Niu

**Affiliations:** 1 Acoustic Science and Technology Laboratory, Harbin Engineering University, Harbin 150001, China; zhaozhishan@hrbeu.edu.cn (Z.Z.); niufang@hrbeu.edu.cn (F.N.); 2 College of Underwater Acoustic Engineering, Harbin Engineering University, Harbin 150001, China; 3 School of Engineering and Technology, University of Hertfordshire, Hatfield AL10 9AB, UK; b.hou@herts.ac.uk (B.H.); r.sotudeh@herts.ac.uk (R.S.)

**Keywords:** adaptive line enhancer, frequency-domain processing, matched filter, time reversal convolution, active sonar detection

## Abstract

The most classical detector of active sonar and radar is the matched filter (MF), which is the optimal processor under ideal conditions. Aiming at the problem of active sonar detection, we propose a frequency-domain adaptive matched filter (FDAMF) with the use of a frequency-domain adaptive line enhancer (ALE). The FDAMF is an improved MF. In the simulations in this paper, the signal to noise ratio (SNR) gain of the FDAMF is about 18.6 dB higher than that of the classical MF when the input SNR is −10 dB. In order to improve the performance of the FDAMF with a low input SNR, we propose a pre-processing method, which is called frequency-domain time reversal convolution and interference suppression (TRC-IS). Compared with the classical MF, the FDAMF combined with the TRC-IS method obtains higher SNR gain, a lower detection threshold, and a better receiver operating characteristic (ROC) in the simulations in this paper. The simulation results show that the FDAMF has higher processing gain and better detection performance than the classical MF under ideal conditions. The experimental results indicate that the FDAMF does improve the performance of the MF, and can adapt to actual interference in a way. In addition, the TRC-IS preprocessing method works well in an actual noisy ocean environment.

## 1. Introduction

The concept of the matched filter (MF) was proposed as early as the period of the Second World War [1]. In electronic information systems of communication, radar, and sonar, the MF is always the most commonly used among a variety of signal detection methods, because the MF is the optimal linear filter that obtains the maximum output signal to noise ratio (SNR) to detect the known signals in white ambient noise. The MF is employed in a wide range of actual applications such as target ranging, channel estimating, and spread spectrum communication.

Dwork [2] gave the expression of the MF in the frequency domain, which will be discussed in detail in Section 2. The appearances of the frequency-domain MF and the fast Fourier transform simplify the implementation of the MF, and make real-time processing possible for it.

The application of the MF in sonar can be traced back to [3,4], which discussed some limitations to the ambiguity function theory of a few common sonar waveforms in terms of their narrow-band approximation, and investigated the echo-to-reverberation power ratio on the output of the MF by using the ambiguity function. These discussions laid the foundation for the application of the MF in sonar detection systems.

Afterwards, many new techniques based on the MF for the application in sonar detection emerged. By way of example, we state four such techniques. The first is the model-based MF [5,6,7], which is generated by correlating the received signal with a reference channel that consists of the transmitted signal convolved with the impulse response of the medium, and, when Doppler compensated, can improve the performance of the MF of the Doppler-shifted signals in a time dispersive ocean environment. The second is the adaptive MF [8,9,10], which is designed to resolve the mismatch problem between the channel model parameters and the actual time-varying and space-varying ocean acoustic channel. The replica of the adaptive MF is adaptively weighted by extracting the time–frequency features of the echo signal with an adaptive filter. The adaptive MF has better matching performance than the model-based MF in slow time-varying coherent-multipath channels. The third technique is the stochastic MF [11,12,13], which can be applied to frequency time-varying signals such as the wide band modulated sonar signal propagated in shallow water. The stochastic MF is able to take into account uncertainties and variations of the multipath channel, thus enhancing the detection efficiency. The fourth technique is the compressive MF [14,15], which is a correlation-based technique that combines analytical techniques in the field of compressive sensing for estimating the unknown delay and amplitude of a received signal using only a small number of noisy, randomly chosen frequency-domain samples of the signal.

Since the MF has been proven to be the optimal detector under ideal conditions, the study of it is mostly under a specific interference background rather than on its own features to improve its performance. Moreover, the better the performance of the detector under ideal conditions, the better the performances of the other improved methods concerning the detector for a specific interference. Is it possible to find a better detector than the MF under ideal conditions? Within the range of linear operation and constant filters, the answer is no. However, jumping out of this range, we propose a frequency-domain adaptive matched filter (FDAMF), which has better expected processing performance.

In this paper, we studied the FDAMF by a theoretical, a simulation, and an experimental method for active sonar detection. We can find a better algorithm than the traditional MF in the field of adaptive processing, because the FDAMF is not a constant filter. It is undoubtedly a great challenge in the field of detection to find a better detector than the optimal one under ideal conditions, and it will also improve the performances of the above methods when combined with them in an actual ocean environment. The FDAMF will provide more reliable and efficient detection techniques to engineering applications in active sonar, radar, and communications.

## 2. Analysis of Output Spectrum of the MF

In this section, we analyze the output spectrum of the MF to prove that the frequency-domain output contains sinusoidal components. In other words, that there are line components in the spectrum of the output spectrum. Thus, we can transform the output spectrum of the MF into a frequency-domain line detection problem. This is the theoretical basis of using an adaptive line enhancer (ALE) to improve the performance of the MF.

### 2.1. Theoretical Basis

**Figure 1:**
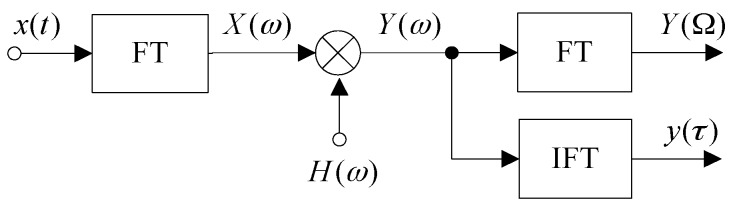
Block diagram of a frequency-domain matched filter (MF).

The block diagram of a frequency-domain MF is shown in Figure 1, in which the FT and the IFT respectively represent the Fourier transform and the inverse Fourier transform.

Assume that the time-domain input signal of the MF is:

(1) x(t)=s(t)+n(t),

where *s*(*t*) is the known specific signal, except the arrival time and amplitude are unknown, and *n*(*t*) is additive zero-mean band-limited white noise. Then, the spectral function of *x*(*t*) can be expressed as:

(2) X(ω)=S(ω)+N(ω),

where *S*(*ω*) and *N*(*ω*) denote the FT of *s*(*t*) and *n*(*t*), respectively, and *ω* = 2*πf*.

The frequency-domain output *Y*(*ω*) of the MF is given by:

(3) $$\begin{array}{cc} Y(\omega ) & =H(\omega )X(\omega )\\ & =H(\omega )S(\omega )+H(\omega )N(\omega )\\ & =S_{o}(\omega )+N_{o}(\omega ) \end{array},$$

where *H*(*ω*) is the transmission function of the MF, that is, the FT of the impulse response *h*(*t*). *S_o_*(*ω*) and *N_o_*(*ω*) denote the output spectrum of *s*(*t*) and *n*(*t*) of the MF, respectively. When the input ambient noise is white, the transmission function of the MF is proved to be a complex conjugation of the input spectrum [16]. Assuming that the output signal component *s_o_*(*t*) obtains the maximum output instantaneous SNR at *t* *=* *t*_0_, we have:

(4) $$H(\omega )=kS^{*}(\omega )e^{-j\omega t_{0}},$$

where *k* is a constant, and * means complex conjugation.

From (3) and (4), it follows that:

(5) $$\begin{array}{l} Y(\omega )=kS(\omega )S^{*}(\omega )e^{-j\omega t_{0}}+kN(\omega )S^{*}(\omega )e^{-j\omega t_{0}}\\ \;=k{\left|S(\omega )\right|}^{2}e^{-j\omega t_{0}}+kN(\omega )S^{*}(\omega )e^{-j\omega t_{0}} \end{array}.$$

The second term in (5) is a random spectrum generated by ambient noise, which is white if the signal bandwidth is wide enough. The first term in (5) is the signal component, which can be seen as a continuous wave (CW) with the variable of frequency and the intensity of $k{\left|S(\omega )\right|}^{2}$.

From (3) and (5), it follows that:

(6) $$S_{o}(\omega )=A(\cos\omega t_{0}-j\sin\omega t_{0}),$$

where $A=k{\left|S(\omega )\right|}^{2}$ is defined as an amplitude function. To see this more clearly, the real part and the imaginary part of the output spectrum are respectively given by:

(7) Re[So(ω)]=Acos(2πt0f)Im[So(ω)]=Asin(2πt0f) .

The above equations indicate that the frequency-domain output of a specific signal processed by the MF is a CW signal with the variable of *f*.

As we know, the time-domain output *Y*(*τ*) of the MF is the IFT of the frequency-domain output *Y*(*ω*). Let us define the spectral spectrum *Y*(Ω) as the FT of *Y*(*ω*):

(8) $$Y(\Omega )=\int_{-\infty }^{\infty }Y(\omega )e^{-j\Omega \omega }d\omega .$$

Then, it follows that:

(9) $$\begin{array}{cc} S_{o}(\Omega ) & =\int_{-\infty }^{\infty }Ae^{-j\omega t_{0}}e^{-j\Omega \omega }d\omega \\ & =\int_{-\infty }^{\infty }Ae^{-j\omega (\Omega +t_{0})}d\omega \; \end{array}.$$

From the above equations, we can see that the real part and the imaginary part of the spectral spectrum *Y*(Ω) each have a line component at the spectral frequency Ω *=* *−t*_0_. Hence, we can use the frequency-domain ALE to enhance the line component, and then improve the output SNR to make weak signals be found easily.

### 2.2. Principle Simulation

**Figure 2:**
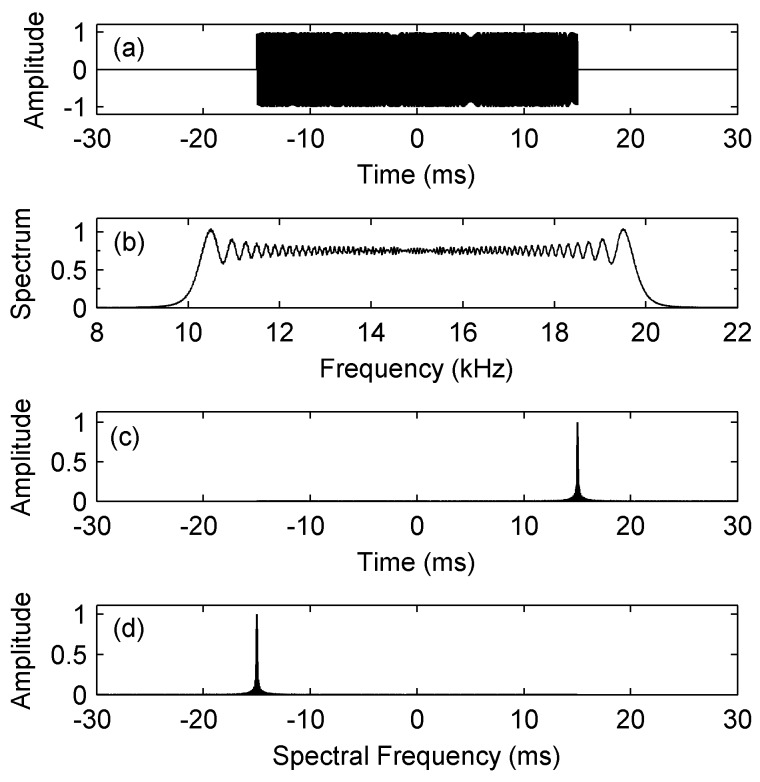
Normalized outputs of the MF. (**a**) The target signal *s*(*t*); (**b**) The output spectrum |Y(ω)|; (**c**) The time-domain output |y(τ)|; (**d**) The spectral spectrum |Y(Ω)|.

In this subsection, we verify the above theoretical analysis by simulation with MATLAB. Assume the target signal *s*(*t*) is a linear frequency modulation (LFM) signal with a starting frequency of 10 kHz, a bandwidth of 10 kHz, and a duration time of 30 ms. As shown in Figure 2a, the starting time of *s*(*t*) is −15 ms and the ending time is 15 ms. Then, the time of the maximum output SNR should be at *t*_0_ = 15 ms, i.e., the end of the signal’s duration, which can be seen in Figure 2c. The output spectrum and the spectral spectrum of the MF are shown in Figure 2b,d respectively. It can be seen that there is a line component at Ω = *−t*_0_ = *−*15 ms.

## 3. Principle of the FDAMF

**Figure 3:**
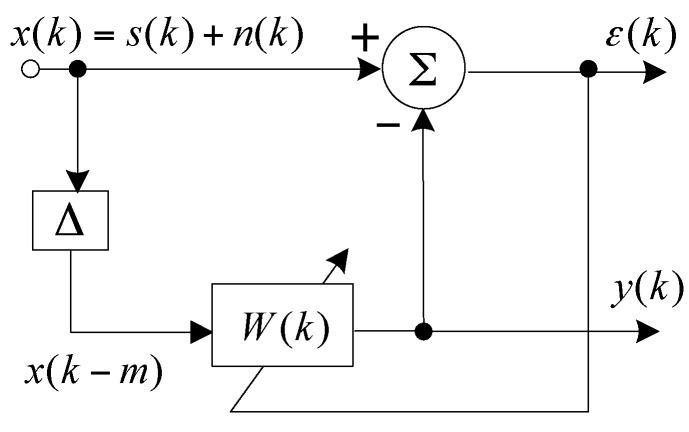
Block diagram of the adaptive line enhancer (ALE).

The block diagram of the time-domain ALE is shown in Figure 3. Δ = *mτ*_0_ is a delay with a sampling period of *τ*_0_ and a delay unit of m. Δ should be larger than the correlation time of ambient noise to make the noise components of *x*(*k* − *m*) and *x*(*k*) uncorrelated. Meanwhile, Δ should be smaller than the correlation time of the signal components to make them correlated [17]. *W*(*k*) is the weight vector calculated by the least mean square (LMS) iterative algorithm, and *ε*(*ω*) is the estimation error to adjust the value of *W*(*k*). In this paper, we apply the time-domain ALE to the frequency-domain by changing the time-variable to a frequency-variable.

**Figure 4:**
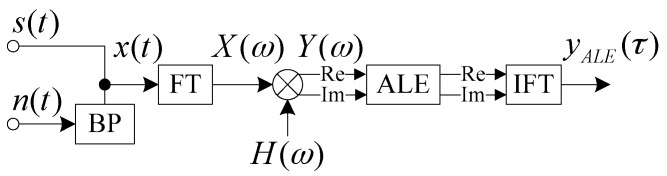
Block diagram of the frequency-domain adaptive matched filter (FDAMF).

An ALE can detect a narrow-band signal or a CW pulse signal with unknown frequency effectively in ambient noise. Because there is a line in the spectral spectrum of the MF, we can use an ALE to process the spectrum *Y*(*ω*) in order to improve the SNR gain. We can then use the IFT to transform the spectrum to the time-domain output *y_ALE_*(*τ*) of the FDAMF, as shown in Figure 4.

**Figure 5:**
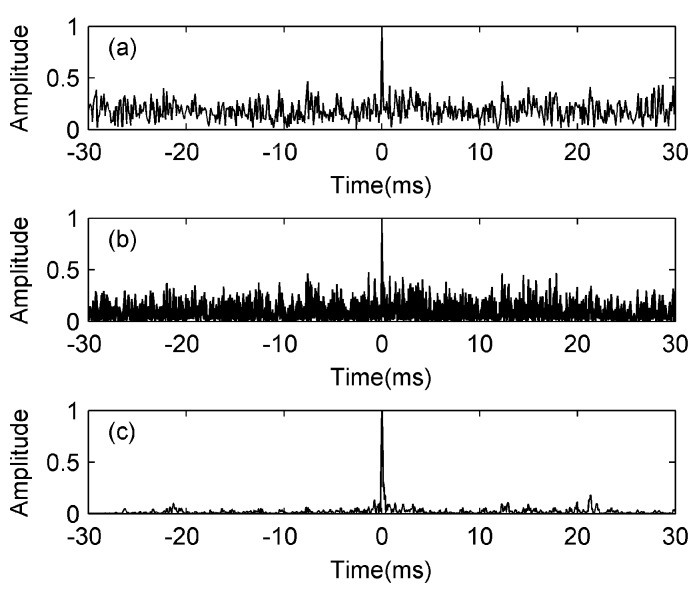
Normalized outputs of the concise fractional Fourier transform (CFRFT), the MF and the FDAMF when input signal to noise ratio (SNR) = −10 dB. (**a**) CFRFT (SNR = 20.2 dB); (**b**) MF (SNR = 21.4 dB). (**c**) FDAMF (SNR = 35.3 dB).

The simulation signal generator is shown in the left parts of Figure 4, in which the BP represents a bandpass filter. The target signal *s*(*t*) is an LFM signal with a starting frequency of 10 kHz, a bandwidth of 10 kHz, and a duration time of 30 ms. The ambient noise *n*(*t*) is zero-mean white noise. The input SNR is −10 dB by taking the noise after passing a bandpass filter (BPF) into account. In this simulation, the FT is carried out by the fast Fourier transform (FFT). We put the ending time of the target signal to the location of 0 ms for easier observation. As this study involves the detection of the LFM signal in noise, we choose an effective detecting tool, the concise fractional Fourier transform (CFRFT) [18], as a comparison to the MF. The output of the CFRFT is shown in Figure 5a. After processing the target signal contaminated by the noise with the block diagrams in Figure 1 and Figure 4 respectively, we can obtain the outputs of the MF and the FDAMF, as shown in Figure 5b,c, in which the step of the ALE is 10^−5^ and the number of weights is 256 (in this paper, the number of weights and the step of the ALE are taken to be these values unless otherwise stated).

From the comparison in Figure 5, we can see that the processing effect of the MF is slightly better than that of the CFRFT, which is in accord with the conclusion that the MF is the optimal detector under ideal conditions. The SNR gain of the FDAMF is significantly higher than that of the MF. The output SNR of the MF is 21.4 dB and that of the FDAMF is 35.3 dB, while the SNR gain of the FDAMF to the conventional MF is 13.9 dB in this simulation.

## 4. Improvement of the FDAMF under Low Input SNR

Although the FDAMF has achieved more obvious SNR gain than the MF in white ambient noise, the ALE processing gain depends on the input SNR, based on the property itself. The ALE processing gain will drop sharply when the input SNR is very low [19]. In order to make the ALE work more efficiently, it is necessary to preprocess the input signals.

As proved above, the frequency-domain output of the target signal processed by the MF is a CW signal with the variable of frequency, whereas the frequency-domain output of the band-limited white noise processed by the MF is still band-limited white. Therefore, we propose a time reversal convolution and interference suppression (TRC-IS) method in the frequency-domain to preprocess the spectrum of the MF to improve the input SNR of the ALE, based on the differences of the spectrums of the target signal and noise processed by the MF, and finally improve the SNR gain of the FDAMF.

Assume that a weak CW signal which is contaminated by additive noise can be expressed as:

(10) x(k)=Acos(ωk+φ)+Bn(k),

where *k* is an integer (1, 2, 3, … , N), and *ω* = 2*πfT_s_*, where *f* denotes frequency and *T_s_* denotes sampling period. *A* and *B* are constants which denote the intensity of the signal and noise.

Expressing the sequence of the samples as a vector, we have:

(11) $$x={\left[x_{1},x_{2},\cdots ,x_{N}\right]}^{T},$$

where *x_k_ = x*(*k*), and the correlation matrix of **x** is **R** = **xx**^T^.

(12) $$R=\left[\begin{array}{cccc} x_{11} & x_{21} & \cdots & x_{N,1}\\ x_{12} & x_{22} & \cdots & x_{N,2}\\ \vdots & \vdots & \ddots & \vdots \\ x_{1,N} & x_{2,N} & \cdots & x_{N,N} \end{array}\right],$$

where *x_i_*_,*j*_ = *x*(*i*)*x*(*j*).

The output of the time reversal convolution (TRC) is a sequence in the order of diagonal direction of matrix **R**, i.e., the TRC converts **R** into a sequence:

(13) $$\begin{array}{l} y(k)=y_{1}+y_{2}+\cdots +y_{N}+\cdots y_{2N-1},\\ where\left\{ \begin{array}{l} y_{1}=x_{1,N}\\ y_{2}=x_{1,N-1}+x_{2,N}\\ \;\cdots \\ y_{N}=x_{11}+x_{22}+\cdots +x_{N,N}\\ \;\cdots \\ y_{2N-1}=x_{N,1} \end{array}\right. \end{array}.$$

The TRC, which contains all of the elements of the correlation matrix, calculates these elements in a simple and fast way, i.e., the TRC contains all of the information of the correlation matrix. In (13), *y_N_* is the auto-correlation term, which has no anti-interference ability. When the SNR is low, *y_N_* mainly contains the energy of the interference, and therefore it should be suppressed.

**Figure 6:**
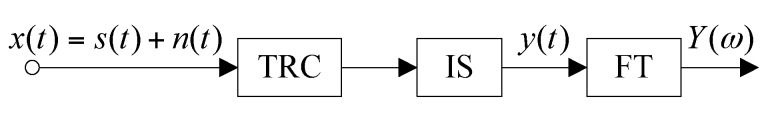
Block diagram of time reversal convolution and interference suppression (TRC-IS).

**Figure 7:**
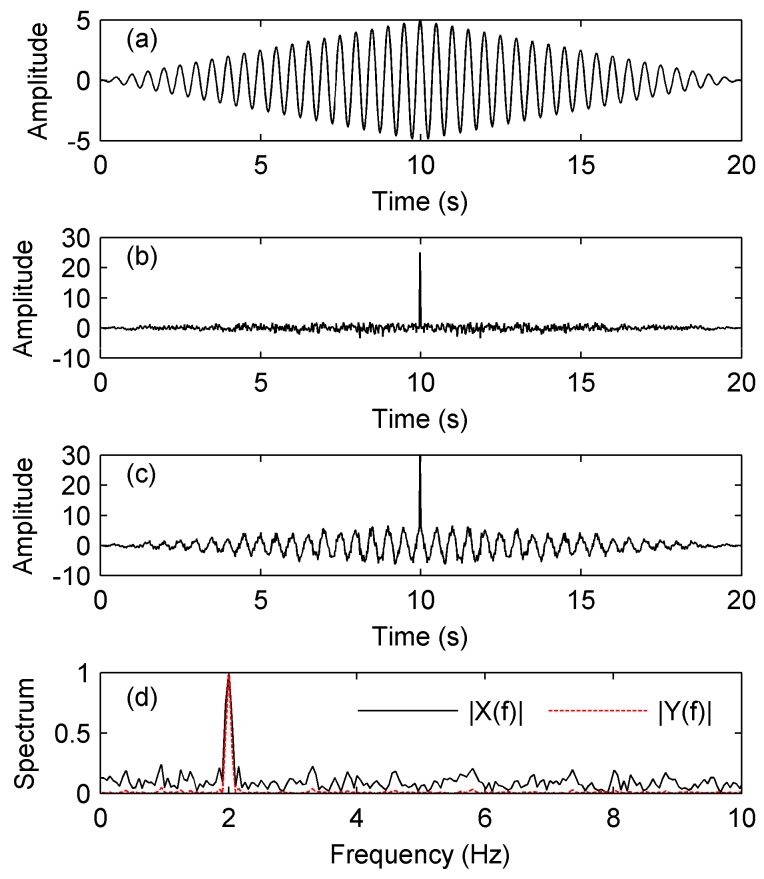
Simulation results of the TRC-IS when the SNR = −10 dB. (**a**) time reversal convolution (TRC) of the signal *s*(*t*); (**b**) TRC of the noise *n*(*t*); (**c**) TRC of the signal contaminated by noise *x*(*t*); (**d**) Normalized spectrum with and without TRC-IS.

The simulation below is based on Figure 6, which shows the block diagram of the TRC-IS. We use low-frequency simulation signals to show the processing effects more clearly. In this simulation, the input signal *s*(*t*) is a CW signal with a frequency of 2 Hz and a bandwidth of 10 s. The noise *n*(*t*) is an additive white Gaussian noise (AWGN). The input SNR is −10 dB. Figure 7a,b show the TRC results of *s*(*t*) and *n*(*t*), from which we can see that the energy of the noise is mainly concentrated at the spike of *t =* 10 s, whereas the energy of the signal is relatively scattered. The result of the signal *x*(*t*) contaminated by noise *n*(*t*) processed by the TRC is shown in Figure 7c; suppressing the interference of which is to set zero to the spike of the noise energy, then we obtain the output of the TRC-IS *y*(*t*). Normalizing the spectrums of *x*(*t*) and *y*(*t*), we get Figure 7d, in which the solid line represents the normalized spectrum of *x*(*t*), and the dashed line represents the normalized spectrum of *y*(*t*), the output SNRs of whom are 20.5 dB and 41.1 dB respectively. From the comparison, it can be seen that the TRC-IS can improve the detecting performance of single-frequency signals.

**Figure 8:**
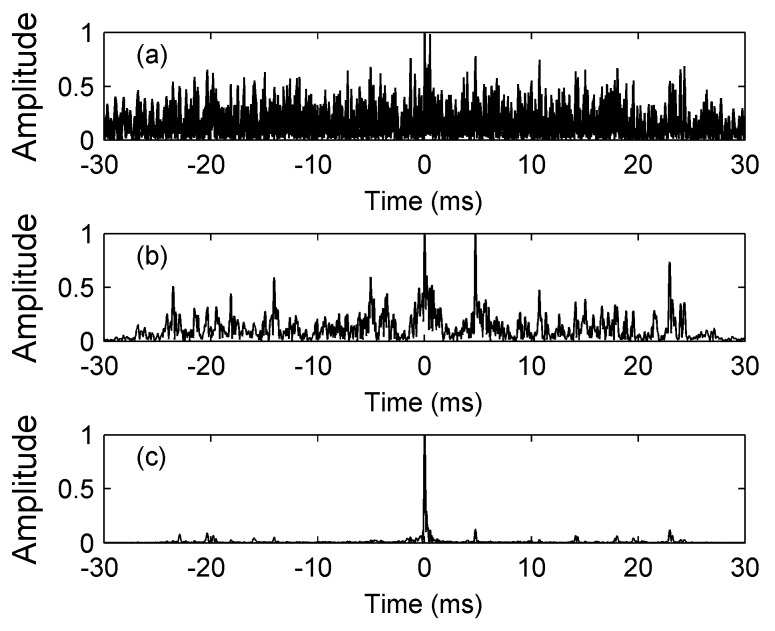
Normalized outputs of the MF, the FDAMF, and the TRC-IS-FDAMF when the input SNR = −15 dB. (**a**) The MF (SNR = 13.3 dB); (**b**) The FDAMF (SNR = 19.5 dB); (**c**) The TRC-IS-FDAMF (SNR = 46.8 dB).

We verify the improving effects of the TRC-IS on the FDAMF in the following simulation. The target signal is an LFM signal with a starting frequency of 10 kHz, a bandwidth of 10 kHz, and a duration time of 30 ms. The ambient noise is a zero-mean AWGN, and the input SNR is −15 dB. The normalized outputs of the MF and the FDAMF are shown in Figure 8a,b, in which the output SNRs are 13.3 dB and 19.5 dB respectively. If we preprocess the input signal of the ALE in Figure 4 with the TRC-IS in Figure 6, we can obtain the TRC-IS-FDAMF output, as shown in Figure 8c, in which the SNR is 46.8 dB. It is seen from Figure 8 that the FDAMF cannot detect useful signals effectively when the input SNR is pretty low, whereas after the TRC-IS preprocessing, improving the input SNR of the ALE, the performance of the FDAMF is improved a lot. Combining the FDAMF with the TRC-IS makes it possible to detect weak signals in strong noise more effectively.

## 5. Performance Analysis

In this section, we use the Monte Carlo method to compare the performance of the MF and the FDAMF. The simulation target signal is an LFM signal with a starting frequency of 10 kHz, a bandwidth of 10 kHz, and a duration time of 30 ms. The interference is a zero-mean AWGN.

**Figure 9:**
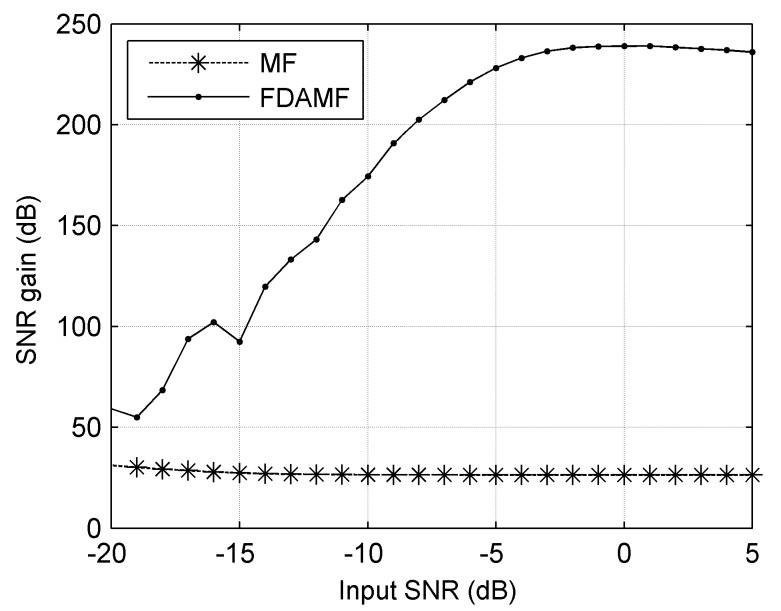
The SNR gain curves of the MF and the FDAMF at different SNRs in additive white Gaussian noise (AWGN).

The SNR gain curves of the MF and the FDAMF at different SNRs in AWGN are shown in Figure 9, in which the number of samples at each input SNR is 1000, and the SNR gain is defined as the difference between the mean of the output SNR and the input SNR. The dashed line denotes the SNR gain of the MF, and the solid line denotes that of the FDAMF. It can be seen that the SNR gain of the FDAMF is significantly higher than that of the MF in the range that Figure 9 shows. With the SNR increasing, the SNR gain of the MF gradually tends to about 26 dB when the input SNR is higher than −10 dB, whereas the SNR gain of the FDAMF increases to about 240 dB. This is in accord with the property of the ALE, i.e., the higher the input SNR the better the performance.

**Figure 10:**
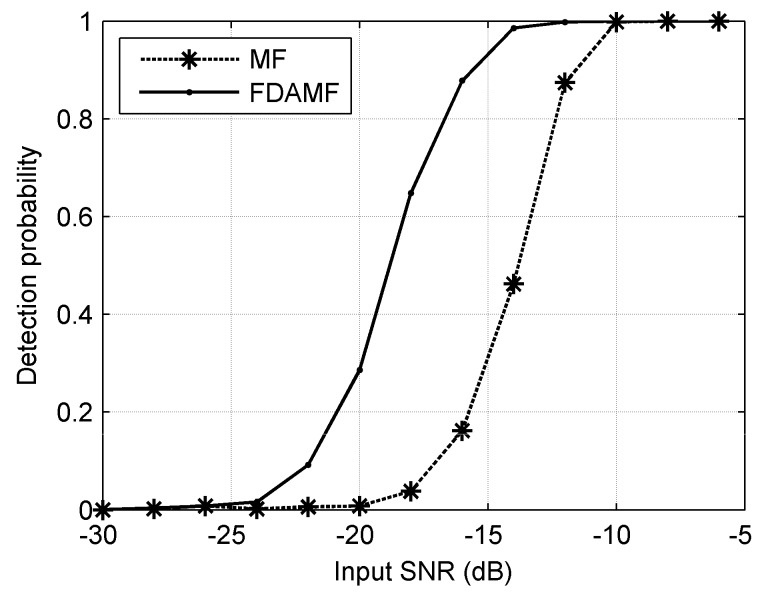
The detection probability curves of the MF and the FDAMF when false-alarm probability is 10^−3^ in AWGN.

Figure 10 shows the detection probability curve when the false-alarm probability is 10^−3^. The dashed line denotes the MF and the solid line denotes the FDAMF. When a specific detection probability is required, such as 0.8, the detection threshold of the FDAMF is about 5 dB lower than that of the MF, i.e., the FDAMF can work more efficiently than the MF at a lower input SNR. When a specific input SNR is required, such as −15 dB, the detection probability of the FDAMF is significantly higher than that of the MF, i.e., the FDAMF is better at detecting targets than the MF in a certain ambient interference.

**Figure 11:**
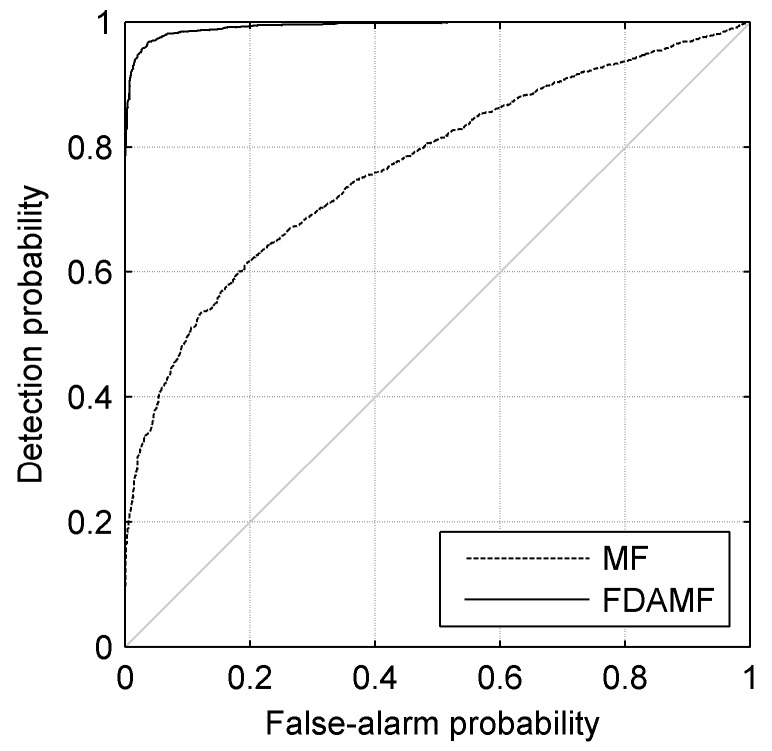
The receiver operating characteristic (ROC) curves of the MF and the FDAMF when SNR = −16 dB in AWGN.

Figure 11 shows the receiver operating characteristic (ROC) curves of the MF and the FDAMF when the input SNR = −16 dB. The dashed line denotes the MF and the solid line denotes the FDAMF. It is seen in Figure 11 that the ROC of the FDAMF is obviously better than that of the MF. When a specific false-alarm probability is required, such as 0.1, the detection probability of the FDAMF can be as high as 0.98, while that of the MF is only 0.50. When a specific detection probability is required, such as 0.8, the false-alarm probability of the FDAMF is less than one thousandth, while that of the MF reaches about 0.48.

## 6. Experimental Results

**Figure 12:**
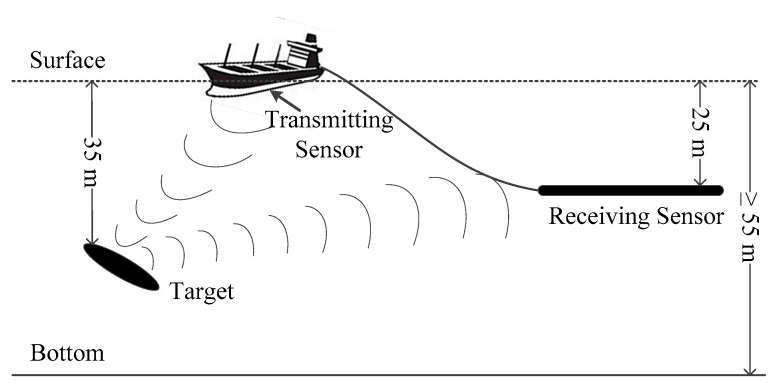
The sea trial installation.

The sea trial installation is shown in Figure 12. The LFM signal is transmitted from the flank array sonar of the surveying ship and received by the towed line array sensor located at a depth of 25 m. The distance between the transmitting sensor and the receiving sensor is about 200 m. The target is about 2 km away from the surveying ship, and is located at a depth of 35 m. The average depth of the sea area is 55 m or more, and the seabed consists of sediment. The sound speed measured in the sea trial was 1470 m/s. We use the data received in this sea trial to verify the performance and practicability of the FDAMF for signal detection.

**Figure 13:**
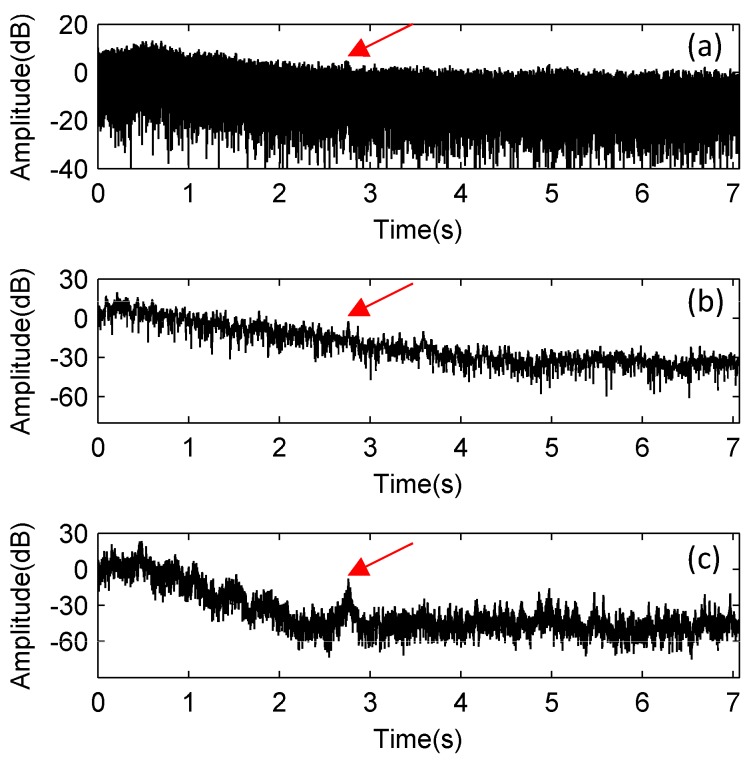
The comparison of the processing results of the received signals from the sea trial: (**a**) Using the MF (SNR = 3 dB); (**b**) Using the FDAMF (SNR = 7 dB); (**c**) Using the TRC-IS-FDAMF (SNR = 16 dB). The arrival time of the target echo is 2.76 s.

The processing results of the received signals from the sea trial by using the MF, the FDAMF, and the TRC-IS-FDAMF respectively are shown in Figure 13. We use logarithmic coordinates to display the results because of the strong interference between 0 s to 1 s and the high fluctuations of the processing results. We can estimate the arrival time of the target echo to be around 2.8 s from the relative position of the transmitting array, the receiving array, and the target. It can be seen from Figure 13a that the target echo is almost submerged in the background interference when processing the received signals with the MF, and the processing results of the FDAMF is also not satisfactory in Figure 13b due to the low SNR, whereas a significant peak of the target echo is observed at 2.76 s of the received signals after processing with the TRC-IS-FDAMF in Figure 13c. The SNR of the received signals can be estimated to be approximately −8 dB according to the position of the target echo. We calculate the output SNR of each method in Figure 13 to be about 3 dB, 7 dB, and 16 dB. So, the SNR gains of the MF, the FDAMF, and the TRC-IS-FDAMF are 11 dB, 15 dB, and 24 dB in this example. The experimental results indicate that the FDAMF does improve the performance of the MF and can adapt to the actual interference in a way. In addition, the TRC-IS preprocessing method works well in very noisy ambient noise.

## 7. Conclusions

In this paper, we proposed an improved MF in AWGN combined with the ALE by analyzing the output spectrum and the spectral spectrum of the MF, which is the FDAMF. When the input SNR is too low, we propose a preprocessing method—TRC-IS—to improve the performance of the FDAMF. We also verified that the performance of the FDAMF is significantly better than that of the MF by simulations and experiments. The methods proposed in this paper can make the detector work more efficiently than the MF at a lower input SNR. It is a great challenge in the field of detection to find a better detector than the optimal one (i.e., the MF) under ideal conditions, and it will provide more reliable and efficient detection technology for the engineering applications in active sonar, radar, and communications. Combined with the appropriate methods, the FDAMF can also be used in actual interferences such as colored noise, reverberation, multipath, and Doppler shift to improve the performance of these methods.
